# Hypopharyngeal Ulcers in COVID-19: Histopathological and Virological Analyses – A Case Report

**DOI:** 10.3389/fimmu.2021.676828

**Published:** 2021-07-05

**Authors:** Andrea Porzionato, Elena Stocco, Aron Emmi, Martina Contran, Veronica Macchi, Silvia Riccetti, Alessandro Sinigaglia, Luisa Barzon, Raffaele De Caro

**Affiliations:** ^1^ Department of Neuroscience, Section of Human Anatomy, University of Padova, Padova, Italy; ^2^ Department of Molecular Medicine, University of Padova, Padova, Italy

**Keywords:** COVID-19, gastrointestinal tract, inflammation, mucosal injury, HSV, histopathology, molecular analysis

## Abstract

In coronavirus disease 2019 (COVID-19), ulcerative lesions have been episodically reported in various segments of the gastrointestinal (GI) tract, including the oral cavity, oropharynx, esophagus, stomach and bowel. In this report, we describe an autopsy case of a COVID-19 patient who showed two undiagnosed ulcers at the level of the anterior and posterior walls of the hypopharynx. Molecular testing of viruses involved in pharyngeal ulcers demonstrated the presence of severe acute respiratory syndrome – coronavirus type 2 (SARS-CoV-2) RNA, together with herpes simplex virus 1 DNA. Histopathologic analysis demonstrated full-thickness lympho-monocytic infiltration (mainly composed of CD68-positive cells), with hemorrhagic foci and necrosis of both the mucosal layer and deep skeletal muscle fibers. Fibrin and platelet microthrombi were also found. Cytological signs of HSV-1 induced damage were not found. Cells expressing SARS-CoV-2 spike subunit 1 were immunohistochemically identified in the inflammatory infiltrations. Immunohistochemistry for HSV1 showed general negativity for inflammatory infiltration, although in the presence of some positive cells. Thus, histopathological, immunohistochemical and molecular findings supported a direct role by SARS-CoV-2 in producing local ulcerative damage, although a possible contributory role by HSV-1 reactivation cannot be excluded. From a clinical perspective, this autopsy report of two undiagnosed lesions put the question if ulcers along the GI tract could be more common (but frequently neglected) in COVID-19 patients.

## Introduction

In late 2019, a novel coronavirus (CoV), namely severe acute respiratory syndrome (SARS)-CoV-2, was identified and classified as responsible of the coronavirus disease 2019 (COVID-19) which, on March 11, 2020, was declared a pandemic by the World Health Organization.

SARS-CoV-2 infection depends on the host cell factors angiotensin-converting enzyme 2 (ACE2), for entry into cells, and transmembrane serine protease 2 (TMPRSS2), for spike (S) protein priming; thus, ACE2 and TMPRSS2 expression levels and distribution likely modulate tissue/organs susceptibility to SARS-CoV-2, including that of the gastrointestinal (GI) tract.

In COVID-19, ulcerative lesions have been reported in various segments of the gastrointestinal (GI) tract, namely the tongue, hard/soft palate, labial and buccal mucosa, [e.g. (1–8)], oropharynx (3), esophagus (9, 10), stomach/duodenum (11, 12) and large bowel (13).

Herein, we report the case of a patient who died of respiratory failure due to COVID-19, whose hypopharynx showed two previously undiagnosed ulcers at autopsy. The lesions were characterized through histopathological/immunohistochemical analyses and molecular biology search for viruses. To the best of our knowledge, this is the first report of hypopharynx ulcer in COVID-19 and the first histopathological and bio-molecular analysis of autopsy samples of a COVID-19-associated ulcerative lesion of the GI tract.

## Case Description

**Figure 1:**
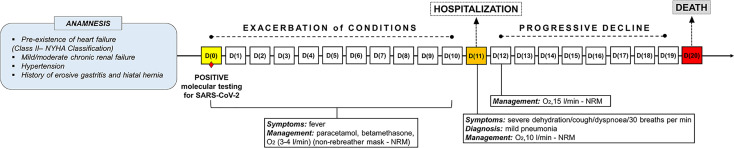
Timeline showing the disease course of the patient from positive molecular testing for SARS-CoV-2 up to death.

An elderly patient was diagnosed as positive for SARS-CoV-2 by molecular testing on nasopharyngeal swab. Pre-existence of heart failure (Class II according to the New York Heart Association – NYHA Classification), mild/moderate chronic renal failure, hypertension and a history of erosive gastritis and hiatal hernia were present in anamnesis. After an initial phase (from 1^st^ to 10^th^ day after the positive swab) characterized by fever and managed with paracetamol, betamethasone and oxygen therapy (3-4 litres/min), a significant clinical exacerbation occurred, requiring hospitalization. Upon admission, on the 11^th^ day of ascertained positivity, the patient showed severe dehydration, cough and dyspnoea (respiratory rate of 30 breaths per min), with diagnosis of mild pneumonia. Oxygen was supplied at 10 liters/min with non-rebreather mask. On the 12^th^ day, oxygen therapy was increased at 15 liters/min. However, a progressive clinical decline occurred. On the 20^th^ day, the patient died of severe respiratory failure (**Figure 1**). Endotracheal intubation was never performed. Signs or symptoms ascribable to the hypopharyngeal ulcers were not ascertained along the clinical course.

**Figure 2:**
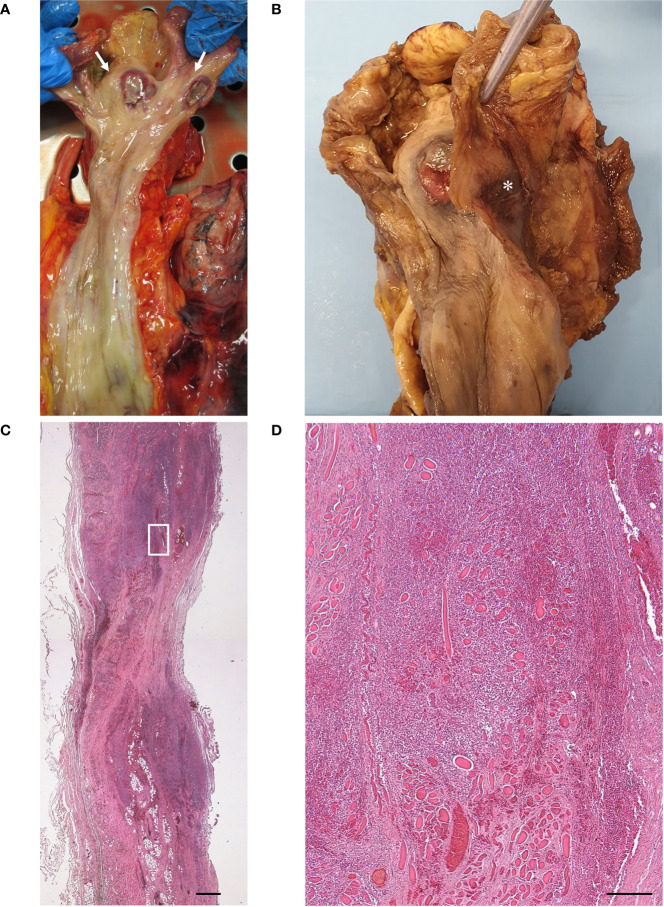
Macroscopic examination of fresh **(A)** and fixed **(B)** hypopharynx and esophagus, with evidence of two necrotic ulcers [white arrows in **(A)**], with raised sharply demarcated borders, in the anterior and posterior hypopharyngeal walls. Note the hemorrhagic infiltration of the external surface of the posterior pharyngeal wall [asterisk in **(B)**]. No lesions were detected in the esophagus. Histopathologic examination of transverse section of the posterior ulcer, with thinning of the pharyngeal wall at the level of the ulcer center and full-thickness inflammatory infiltration extending beyond the lesion margins **(C)** (scale bar 800 µm). Higher magnification of **(C)** insert, showing inflammatory infiltration of the muscle layer with necrosis and degeneration of the skeletal muscle fibers **(D)** (scale bar 200 µm).

At autopsy, two necrotic ulcerations with overlying fibrin clot (about 1.5 cm in diameter) were found at the level of the anterior and posterior walls of the hypopharynx (**Figures 2A, B**). The anterior lesion was located on the pharyngeal wall covering the lamina of the cricoid cartilage. In correspondence of the posterior ulcer, the external surface of the pharyngeal wall also showed hemorrhagic infiltration (**Figure 2B**). Full-thickness samples of the above lesions were collected. There were no other ulcers or erosions in the other segments of the GI tract nor in the laryngeal and trachea-bronchial mucosa. The lungs showed pulmonary edema and congestion, with sparse aspects of consolidations; multiple thromboses in medium-sized vessels were also found.

Real-time PCR analyses were performed to detect viruses involved in oropharyngeal ulcers, i.e., herpes simplex virus type 1 (HSV-1), herpes simplex virus type 2 (HSV-2), varicella zoster virus (VZV), Epstein-Barr virus (EBV), and enteroviruses, in addition to SARS-CoV-2. Briefly, total RNA and DNA was purified from paraffin-embedded 20 µm thick sections using a RecoverAll™ Total Nucleic Acid Isolation kit (Thermo Fisher Scientific, Waltham, MA, USA) following the manufacturer’s instructions. Real-time PCR analyses were performed by using primers and TaqMan probes as reported (14–17) and run on ABI 7900HT Sequence Detection Systems (Thermo Fisher Scientific).

Immunoperoxidase staining was performed on a Dako EnVision Autostainer according to manufacturer recommendations. Antibodies for CD3 (Polyclonal Rabbit Anti-Human, Dako Omnis, Code Number: GA503), CD20 (Monoclonal Mouse Anti-Human, Clone KP1, Dako Omnis, Code Number: M0814),

CD61 (Monoclonal Mouse Anti-Human, Clone Y2/51, Dako Omnis, Code Number: M0753) and CD68 (Monoclonal Mouse Anti-Human, Clone L26, Dako Omnis, Code Number: M0756) were employed to characterize lympho-monocytic infiltration and evaluate the presence of platelet microthrombi, while anti-SARS-CoV-2 Spike Subunit 1 Antibody (Monoclonal Rabbit Anti-Human, Clone 007, Sino Biological, Code Number: 40150-R007) was employed to evaluate viral tropism within the tissue. Spike antibody was validated employing SARS-CoV-2 infected Vero E6 cells and autopsy-derived lung tissue from SARS-CoV-2 positive patients as positive controls, while non-infected cells and lung sections deriving from autopsy cases predating COVID-19 pandemic (2017) were used as negative controls. A non-COVID-19 ulcer (an ulcer by *Aspergillus fumigatus* in a neoplastic patient) was also employed as control, to evaluate possible non-specific staining for necrotic tissue. Additionally, immunohistochemistry for HSV (Anti-ICP27, H1142, Santa Cruz) was performed. Peroxidase reactions were repeated at least three times to ensure reaction consistency. Slides were evaluated by experienced morphologists and disagreements were resolved by consensus.

Molecular analyses detected SARS-CoV-2 RNA and HSV-1 DNA in both hypopharyngeal ulcers, while testing for HSV-2, VZV, EBV and enteroviruses gave negative results.

**Figure 3:**
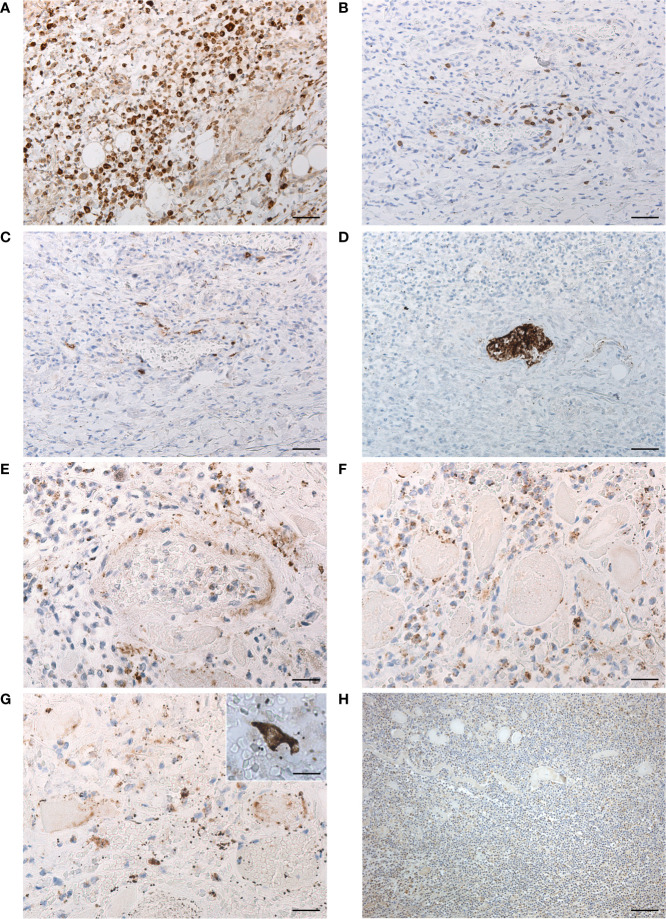
Characterization of lympho-monocytic infiltration **(A–C)** through anti-CD68 **(A)**, -CD3 **(B)** and -CD20 **(C)** immunostaining (scale bars 60 µm). Anti-CD61 immunostaining with evidence of platelet microthrombus **(D)** (scale bars 60 µm). Immunohistochemistry for SARS-CoV-2 Spike Subunit 1 **(E–G)**, showing positive cells in perivascular infiltration **(E)** (scale bar 30 µm), among necrotic skeletal muscle fibers **(F)** (scale bar 30 µm) and in the deep lamina propria **(G)** (scale bar 30 µm), with detail of another positive cell in insert (scale bar 20 µm). Immunohistochemistry for HSV1 **(H)**, showing general negativity of inflammatory infiltration of the muscle layer, except for some rare positive cells (scale bar 120 µm).

Histopathologic analysis of the ulcer showed thinning of the pharyngeal wall, due to necrosis/disruption of the mucosal layer and deep skeletal muscle fibers (**Figures 2C, D**). Extensive lympho-monocytic infiltration and multiple hemorrhagic foci involved all the pharyngeal layers, also beyond the ulcer margins. Lympho-monocytic component was mainly composed of CD68-positive elements (**Figure 3A**); CD3- (**Figure 3B**) and CD20- (**Figure 3C**) positive lymphocytes were also detected. Some small vessels were occluded by fibrin and platelet microthrombi, the presence of the latter being confirmed by anti-CD61 immunostaining (**Figure 3D**). SARS-CoV-2-positive cells were also recognized throughout the thickness of the pharyngeal wall, from the ulcerated mucosa to the deep muscle layer (**Figures 3E–G**). Lympho-monocytic cells expressing the SARS-CoV-2 Spike Subunit 1 were also specifically identified in the context of the inflammatory infiltrations disposed around muscle fibers and blood vessels. Immunohistochemistry for HSV1 showed general negativity for inflammatory infiltration, although in the presence of some positive cells (**Figure 3H**). The non-COVID-19 ulcer was totally negative at immunohistochemistry for SARS-CoV-2 Spike Subunit 1 (**Supplementary Figures 1A, B**).

**Figure 4:**
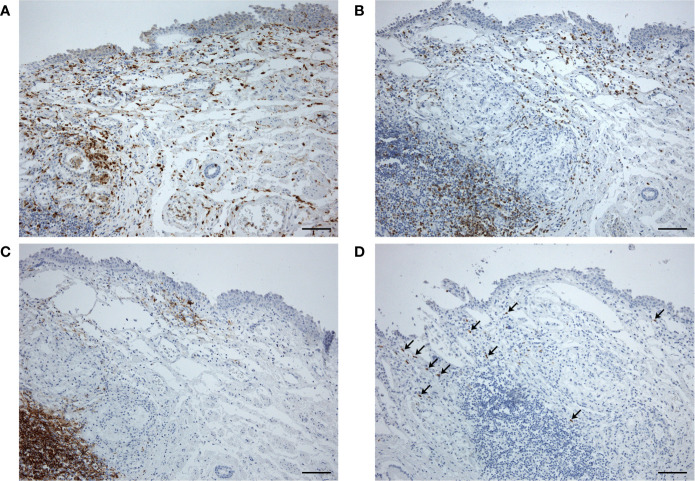
Esophageal sample at distance from the hypopharyngeal ulcer, showing moderate lympho-monocytic infiltration (**A**, anti-CD68; **B**, anti-CD3; **C**, anti-CD20) and some cells immunostained for SARS-CoV-2 Spike Subunit 1 (**D**, arrows) (scale bars 120 µm).

At microscopic examination of the GI tract, some moderate lympho-monocytic infiltrations were also present in the esophageal mucosa (**Figures 4A–C**), in the presence of cells positive for SARS-CoV-2 Spike Subunit 1 (**Figure 4D**).

The lung histopathology (**Supplementary Figures 1C-E**) was characterized by diffuse alveolar damage, vascular congestion, platelet/fibrin thromboses in small- to medium-sized vessels, alveolar and subpleural haemorrhagic infiltrations, and lymphomonocytic infiltrations. Immunohistochemistry for SARS-CoV-2 Spike Subunit 1 showed multiple positive cells. Real-time PCR on paraffin-embedded sections from lung samples confirmed positivity for SARS-CoV-2. Multiple microthromboses were also found in esophageal mucosa, spleen, kidney, adrenal gland and brainstem (**Supplementary Figure 1F**). Focal lympho-monocytic infiltrations were also found in liver and adrenal glands.

## Discussion

This case report focuses on presence of undiagnosed hypopharynx ulcerative lesions in a SARS-CoV-2 positive patient deceased after exacerbation of COVID-19. In the broad and multifaceted scenario characterizing SARS-CoV-2 infection, different types of lesions have been recognized, proving the polymorphic nature of the virus-associated manifestations. Although non-pulmonary tissues are recognized as susceptible target to SARS-CoV-2, their effective involvement in COVID-19 requires further examination.

Ulcerative lesions have been clinically reported in the GI tract of COVID-19 patients; however, only few authors addressed histopathology of the lesions. In particular, histopathological analyses have been performed on oral ulcer biopsies in SARS-CoV-2 positive patients (2, 5). Soares et al. (5) reported diffuse chronic inflammatory infiltrates, focal necrosis and hemorrhages, together with thrombosis of superficial and deep small vessels. Inflammatory infiltration was mainly composed of CD3- and CD8-positive lymphocytes. Ansari et al. (2) described edema, mucosal desquamation, granulation and ulceration, with invasion of mononuclear cells characterized by large and glassy nuclei. They also reported neutrophilic infiltration due to secondary bacterial infection. Lymphocytic infiltration has also been described in biopsy of a COVID-19-associated esophageal ulcer (10). Specific immunohistochemical analyses for SARS-CoV-2 were not performed in the above cases.

With respect to the above previous reports, we here describe an autopsy case of undiagnosed ulcer of the GI tract in a COVID-19 patient. To our knowledge, no similar cases have been reported so far in the literature. The full-thickness and large sampling allowed for a better evaluation of the microscopic findings with respect to incisional biopsies. In particular, we described an extensive lympho-monocytic infiltration of all the pharyngeal layers, from the mucosa to the external aspect of the muscle layer, with multiple foci of hemorrhagic infiltration, also macroscopically visible on the external surface of the posterior pharyngeal wall (**Figure 2B**). Deep inflammatory infiltration extended also beyond the ulcer margins and it was mainly composed of CD68-positive macrophages, although also CD3- and CD20-positive lymphocytes were present. It is particularly intriguing that inflammation was accompanied by necrosis of the mucosa, completely detached in the ulcer, but also of the skeletal muscle fibers, which were almost totally absent at the center of the lesion. The full-thickness involvement of the ulcer has not been previously reported (probably due to the intrinsic superficial nature of the incisional biopsy) and is particularly important also for its clinical implications (higher risk of bleeding and perforation). Moreover, consistently with effects of SARS-CoV-2 in various tissues, multiple fibrin and platelet microthrombi were found in small vessels of the pharyngeal wall. In the present study, it was also possible for the first time to identify SARS-CoV-2 in a GI ulcer not only by Real-time PCR but also through immunohistochemistry. In particular, positive cells were found throughout the thickness of the pharyngeal wall, from the ulcerated mucosa to the deep muscle layer; conversely, the epithelium at the borders of the lesions did not show positive elements for SARS-CoV-2 by immunostaining. The full-thickness pattern of inflammation and necrosis, together with multiple microthromboses and haemorragic aspects, also suggests a probable route of spread from the deep layers, more than epithelium. Ischaemic/haemorrhagic mechanisms probably played a major role in the pathogenesis of the ulcerative lesions.

In the present report, we also detected HSV-1 DNA and immunostained cells in the ulcerative tissue, raising the question about its potential contributory role. Histopathological characteristics of herpetic ulcers include the presence in the epithelium of multinucleated giant cells with eosinophilic intra-nuclear inclusions (Cowdry type A) and nuclear chromatin with ground-glass appearance [e.g (18, 19)]. Conversely, we did not find these findings nor in the ulcerated tissue nor in the close not-ulcerated epithelium. Moreover, herpes simplex esophagitis usually involves the mid- and distal esophageal segments [e.g. (18, 19)]. Thus, our microscopic/immunohistochemical findings and the comparative evaluation of histopathology of HSV-1- and SARS-CoV-2-mediated damages strongly support direct invasion by SARS-CoV-2 as main damage mechanism. However, although the histopathological characteristics of the ulcers do not support a specific etiopathogenetic role by HSV-1, we cannot exclude that SARS-CoV-2 infection could have produced the local damage also through contributory role by HSV-1 reactivation. In fact, reactivation of an oral HSV-1 has also been reported in an intubated COVID-19 patient (6).

In conclusion, our autopsy report of undiagnosed pharyngeal ulcers supports the hypothesis that these lesions in the GI tract of COVID-19 patients could be more common than thought, although frequently neglected. The possibility of such pharyngeal lesions should be also kept in mind for the potential additional risk of iatrogenic injury (bleeding? perforation?) during intubation procedures.

## Data Availability Statement

The original contributions presented in the study are included in the article/**Supplementary Material**. Further inquiries can be directed to the corresponding author.

## Ethics Statement

Ethical review and approval were not required for the study on human participants in accordance with the local legislation and institutional requirements. Written informed consent for participation was not required for this study in accordance with the national legislation and the institutional requirements.

## Author Contributions 

RDC and AP performed autopsy, identified the case, sampled the tissues and were responsible of histopathology and immunohistochemistry findings interpretation and clinical correlation. LB, AS, and SR performed molecular analyses for viruses detection and interpreted the data. AE and MC performed the histological and immunohistochemical analyses. ES, AP, and VM conducted the literature search. All authors contributed to data interpretation. AP, ES, and AE prepared the first draft of the manuscript. RDC, AP, LB, and VM performed the final supervision of the manuscript. All authors contributed to the article and approved the submitted version.

## Funding

This research was funded in part by the European Union’s Horizon 2020 research and innovation programme, under grant agreement no. 874735 (VEO).

## Conflict of Interest

The authors declare that the research was conducted in the absence of any commercial or financial relationships that could be construed as a potential conflict of interest.

## Supplementary Material

Supplementary Figure 1 A non-COVID-19 ulcer as negative control for immunohistochemistry anti-SARS-CoV-2 Spike Subunit 1 **(A, B)** (scale bars 120 µm). Lung representative histopathology **(C)** and multiple positive cells at immunohistochemistry for SARS-CoV-2 Spike Subunit 1 in lung sample **(D)** (scale bars 120 µm). CD61-positive thrombosis of small vessels of lung **(E)** and brainstem **(F)** (scale bars 60 µm).
