# Supplementary figures and images for: Hypopharyngeal Ulcers in COVID-19: Histopathological and Virological Analyses – A Case Report
Source: Front Immunol. 2021 Jul 5;12:676828. doi: 10.3389/fimmu.2021.676828 (PMC8287416; doi:10.3389/fimmu.2021.676828)

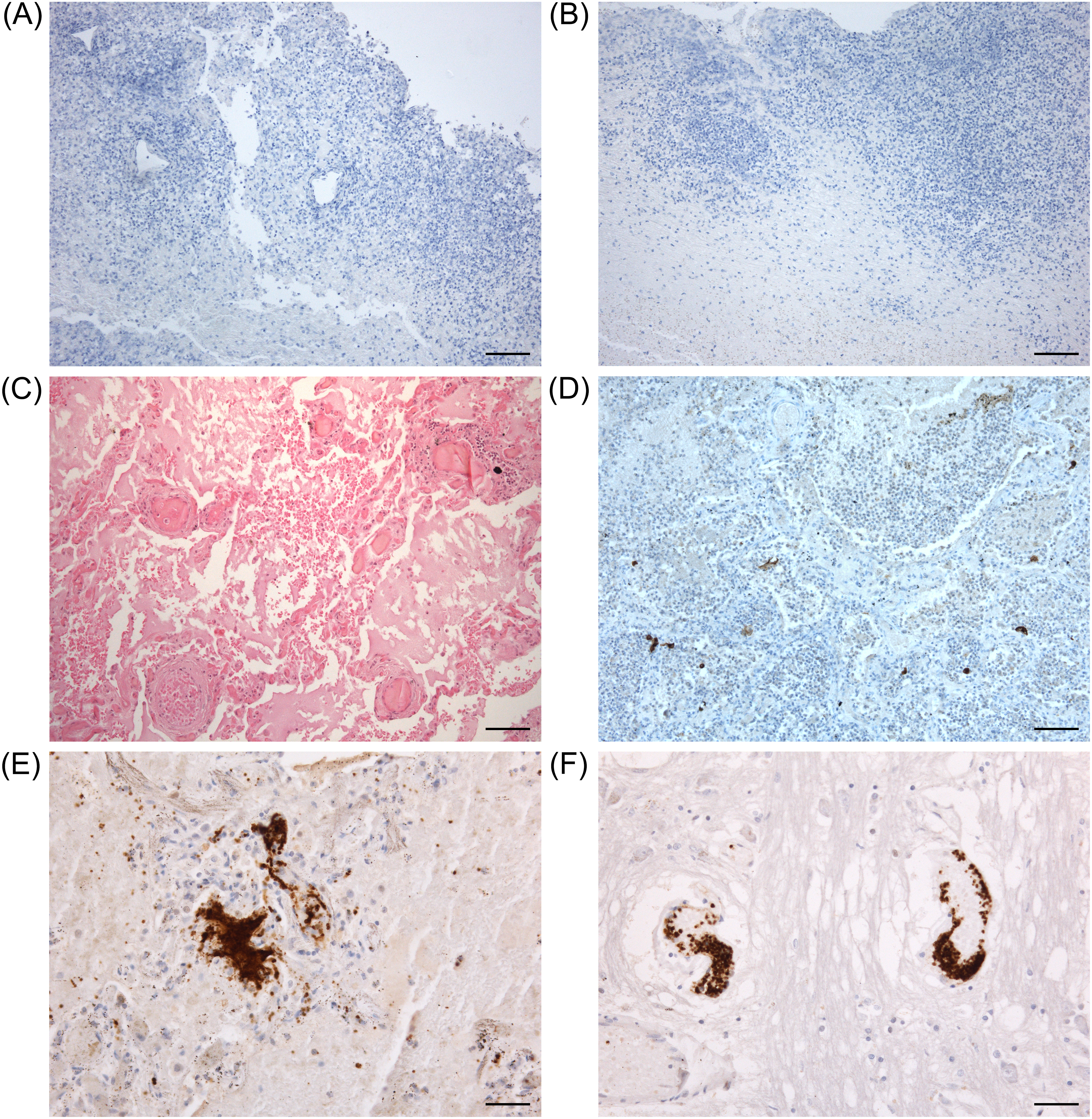

Supplement: Supplementary Figure 1 — A non-COVID-19 ulcer as negative control for immunohistochemistry anti-SARS-CoV-2 Spike Subunit 1 (A, B) (scale bars 120 µm). Lung representative histopathology (C) and multiple positive cells at immunohistochemistry for SARS-CoV-2 Spike Subunit 1 in lung sample (D) (scale bars 120 µm). CD61-positive thrombosis of small vessels of lung (E) and brainstem (F) (scale bars 60 µm). [file Image_1.tif]
